# Effect on longitudinal growth and anemia of zinc or multiple micronutrients added to vitamin A: a randomized controlled trial in children aged 6-24 months

**DOI:** 10.1186/1471-2458-10-145

**Published:** 2010-03-18

**Authors:** Meera K Chhagan, Jan Van den Broeck, Kany-Kany A Luabeya, Nontobeko Mpontshane, Andrew Tomkins, Michael L Bennish

**Affiliations:** 1 Department of Paediatrics and Child Health, University of KwaZulu-Natal, Umbilo Road, Durban, South Africa; 2 Friedman School of Nutrition Science and Policy, Tufts University, Harrison Avenue, Boston, USA; 3 Department of Epidemiology and Public Health, University College Cork, College Road, Cork, Ireland; 4 South African Tuberculosis Vaccine Initiative, Institute of Infectious Diseases & Molecular Medicine, University of Cape Town, Cape Town, South Africa; 5 School of Child & Adolescent Health, University of Cape Town, Cape Town, South Africa; 6 Department of Clinical Development, Pharmaceutical Product Development, Granta Park, Cambridge, UK; 7 Centre for International Health and Development, Institute of Child Health, University College, London, WC1N 1EH, UK; 8 Mpilonhle, Mtubatuba, 3935, South Africa; 9 Department of Population, Family and Reproductive Health, Bloomberg School of Public Health, John Hopkins University, Wolfe Street, Baltimore, USA

## Abstract

**Background:**

The benefits of zinc or multiple micronutrient supplementations in African children are uncertain. African children may differ from other populations of children in developing countries because of differences in the prevalence of zinc deficiency, low birth weight and preterm delivery, recurrent or chronic infections such as HIV, or the quality of complementary diets and genetic polymorphisms affecting iron metabolism.

The aim of this study was to ascertain whether adding zinc or multiple micronutrients to vitamin A supplementation improves longitudinal growth or reduces prevalence of anemia in children aged 6-24 months.

**Methods:**

Randomized, controlled double-blinded trial of prophylactic micronutrient supplementation to children aged 6-24 months. Children in three cohorts - 32 HIV-infected children, 154 HIV-uninfected children born to HIV-infected mothers, and 187 uninfected children born to HIV-uninfected mothers - were separately randomly assigned to receive daily vitamin A (VA) [n = 124], vitamin A plus zinc (VAZ) [n = 123], or multiple micronutrients that included vitamin A and zinc (MM) [n = 126].

**Results:**

Among all children there were no significant differences between intervention arms in length-for-age Z scores (LAZ) changes over 18 months. Among stunted children (LAZ below -2) [n = 62], those receiving MM had a 0.7 Z-score improvement in LAZ versus declines of 0.3 in VAZ and 0.2 in VA (P = 0.029 when comparing effects of treatment over time). In the 154 HIV-uninfected children, MM ameliorated the effect of repeated diarrhea on growth. Among those experiencing more than six episodes, those receiving MM had no decline in LAZ compared to 0.5 and 0.6 Z-score declines in children receiving VAZ and VA respectively (P = 0.06 for treatment by time interaction). After 12 months, there was 24% reduction in proportion of children with anemia (hemoglobin below 11 g/dL) in MM arm (P = 0.001), 11% in VAZ (P = 0.131) and 18% in VA (P = 0.019). Although the within arm changes were significant; the between-group differences were not significant.

**Conclusions:**

Daily multiple micronutrient supplementation combined with vitamin A was beneficial in improving growth among children with stunting, compared to vitamin A alone or to vitamin A plus zinc. Effects on anemia require further study.

**Trial registration:**

This study is registered with ClinicalTrials.gov, number .NCT00156832.

## Background

An estimated 12% of South African children below five years of age are underweight, and 25% are stunted[1]. Additionally, a high prevalence of vitamin and mineral deficiency exists in infants and young children, and surveys have found a 21% prevalence of anemia [2-4]. Despite this, a recent trial of multiple micronutrients in infants aged six to twelve months was unable to demonstrate an effect on length-for-age Z-scores, with supplemented children continuing to show a decline in Z-scores over time[5]. The effect of prophylactic zinc and/or additional micronutrients on growth in this population remains to be fully defined.

Zinc supplementation is known to improve growth in certain populations of children. A meta-analysis of zinc supplementation trials found significant beneficial effects on height in children, with the largest benefit occurring in children with low height Z-scores at baseline[6]. Notably, none of the three trials from African countries included in the meta-analysis of effect on height found an improvement in height velocity with zinc supplementation. This may be related to lesser degrees of stunting in the African populations compared to Asian populations where the majority of studies finding benefit were conducted. A more recent trial in Burkina Faso, conducted after the meta-analysis was conducted, found no benefit of zinc on height in infants, even in the subgroup of stunted infants[7]. This raises the question of whether concurrent deficiencies of other micronutrients limited the response to zinc. The previous South African study of infants aged six to twelve months, which failed to find an effect on length-for-age Z-scores, did not examine effects in stunted children[5]. Further investigation is therefore needed to assess the benefits of multiple micronutrient supplementation in African children, especially those at highest risk of growth faltering such as those living in rural areas and those at risk of repeated diarrheal illness.

Anemia in infants and children is multifactorial in origin. While iron deficiency is the most common cause, deficiencies in other nutrients that contribute to iron homeostasis - riboflavin, folic acid, vitamins B12, C and A - may also result in anemia[8]. The high phytate content of weaning diets such as maize porridge, the staple food for rural South African children, may further impair absorption of iron and zinc. Infectious disease, especially chronic infection, is known to alter iron metabolism. This multifactorial nature of anemia may partly explain the incomplete response of anemia to iron supplementation in some settings[9,10]. Some trials of multiple micronutrients, including the combination of iron and zinc, have found them more effective in reducing anemia prevalence than iron alone, [4,9,11,12] while others have not found a beneficial effect of combined supplementation[13]. Because of these discordant results, the utility of zinc or multiple micronutrients in preventing anemia or augmenting its treatment needs further investigation.

In HIV-infected children early viremia and subsequent disease progression are superimposed on the other factors that contribute to growth faltering and anemia in uninfected children. Poor longitudinal growth becomes apparent early in infancy. Unlike weight, height fails to respond to increased feeding[14]. It is conceivable that infected children may show a suboptimal response to supplements due to altered metabolism of these nutrients and reduction in erythropoiesis [15] or the direct effect of HIV itself[16].

We analyzed data from a randomized, controlled, double-blinded community-based trial whose primary aims were to assess the benefit of prophylactic daily zinc or multiple micronutrient supplements, both combined with vitamin A, in comparison to vitamin A alone that was used as a control group, on: 1) the primary prevention of respiratory illness and diarrhea, and 2) the effect on growth in rural South African children[17]. Supplements used in this trial had no overall effect on prevalent days or incidence of diarrhea compared to vitamin A that was used as a control group, [17] but they reduced the incidence of diarrhea in stunted children[18].

The main purpose of this analysis is to report on the second study aim - effect of supplementation on growth - and to report exploratory analyses of the effect of supplementation on anemia. More specifically, the second aim of this trial was to compare the effect on longitudinal length-for-age Z-scores of adding either zinc alone or in combination with multiple micronutrients to vitamin A supplements, and to describe how HIV, stunting and diarrheal morbidity affect the longitudinal growth response.

## Methods

### Study site, population and design

The study was a randomized, controlled double-blinded community-based trial of prophylactic zinc supplements, or multiple micronutrient supplements including zinc, given to three cohorts of children starting at age 6 months and continuing until age 24 months[17]. The trial was performed in a non-malaria endemic area in rural northern KwaZulu-Natal Province, South Africa during 2003 to 2006. Most infants in this area received breast and complementary feeding early in infancy[19]; in the study sample we found high consumption of breast-milk and complex carbohydrates (cereals/maize) between six and nine months of age, with steady declines in breastfeeding thereafter [20].

Details of the study methodology and procedures, and characteristics of this community, have been previously published [17,21]. Briefly, 373 infants were enrolled between June 2003 and October 2004, with follow-up of enrolled children ending in January 2006. Children were excluded from enrolment if they received micronutrient supplements in the previous month, if weight was below 60% of the median for age using NCHS references, [22] if they had nutritional edema, if they had diarrhea for more than seven days at the time of study enrollment, or if co-enrolled in other intervention trials. Randomization was performed after HIV status was determined. For children HIV testing was done with a quantitative HIV RNA assay (Nuclisens HIV-1 QT, Organon Teknika or Nuclisens EasyQ HIV-1, Biomerieux, Boxtel, The Netherlands). For maternal HIV status two ELISA tests were done (first Vironostika HIV-1 Microelisa system and then Uni-Form II plus O if the first test was positive, both Biomèrieux) each of which had to be positive for HIV infection to be diagnosed.

An allocation list was prepared using computer-generated random numbers and a block size of six. The manufacturer prepared numbered packs of tablets corresponding to the allocation list. Assignment to the three treatment arms was done separately for three cohorts of children stratified by HIV status of child and mother: HIV-infected children of HIV-infected mothers; HIV-uninfected children born to HIV-infected mothers; and HIV-uninfected children of HIV-uninfected mothers. Investigators, study staff and participants were blind to the treatment assignments.

None of the children received antiretroviral therapy because of the unavailability of therapy for children in both public and private clinics during the study period. When antiretroviral therapy for children became available in clinics in the area in January 2005, all children with HIV infection in the study were referred for evaluation and initiation of therapy as appropriate.

### Micronutrient supplements

There were three supplement arms, one of which contained only 1250 IU of vitamin A. This served as the control group instead of a placebo arm because the standard of care in South Africa at the time of the study was six-monthly vitamin A supplementation to children below the age of five years. The daily dose of vitamin A used in the trial was equivalent to the 6-monthly high-dose supplementation. The other two supplements contained 10 mg of zinc as zinc gluconate in addition to 1250 IU vitamin A. The multiple micronutrient additionally contained 0.5 mg each of vitamins B1, B2 and B6; 0.9 μg vitamin B12; 35 mg vitamin C; 5 μg vitamin D; 6 mg vitamin E; 10 μg vitamin K; 0.6 mg copper as cupric gluconate; 150 μg folate; 50 μg iodine; 10 mg iron as ferrous fumarate; and 6 mg niacin. All supplements were packaged in identical blister packs of seven tablets. All trial supplement tablets had the same color, form, texture and taste. Caregivers were instructed to crush the tablets into a small volume of porridge and ensure that the entire prepared portion was consumed. Trained field workers made weekly home visits to monitor compliance and to supply study supplements. At each visit, the field workers observed preparation and administration of the tablet. Any tablets remaining from the child's previous supply were counted and recorded. Use of any other micronutrient supplements during the study was documented[17]. Children who were found to have hemoglobin below 10 g/dL during the routine testing performed on all study participants were given therapeutic iron as per South Africa Department of Health guidelines, which recommend 6 mg/kg elemental iron daily in divided doses with meals and continued for three months after hemoglobin level reaches 10 g/dL.

### Procedures

Length, head circumference, mid-upper arm circumference and triceps skin-fold thickness were recorded by a trained study nurse at enrolment and subsequently at scheduled visits to the study clinic. Caregivers and infants were asked to return for scheduled visits at 7, 8, 9, 12, 15, 18, 21 and 24 months of age to assess growth using anthropometry[17]. Nurses were trained on standardized anthropometric measurements according to the guidelines of the Dutch Growth Foundation[23]. An electronic digital scale was used to measure weight to the nearest 0.1 kg. A Harpenden infantometer was used to measure recumbent length to the nearest 0.1 cm (Holtain, Crymych, UK). Intra-observer and inter-observer technical errors of measurement for anthropometric data were previously reported for this trial and showed acceptably low measurement error rates[21]. Hemoglobin determination was done at the clinic by study nurses trained in the standardized use of a portable HemoCue system (Angelholm, Sweden) that is also routinely used for measuring hemoglobin in health services. Information on diarrhoea was collected during weekly home visits[17].

### Growth analyses

Length-for-age and weight-for-age Z-scores for each child at each visit were computed using the WHO Child Growth Standards[24]. A changing trend in Z-score over time may represent growth faltering or catch-up growth given the child's preceding Z-score. For growth assessment the outcome measure was change in length-for-age Z-score over 18 months. The main predictor included in the analysis was treatment group. We used mixed effects regression models using PROC MIXED in SAS V9.1 (SAS Institute, Inc., Cary, North Carolina) to construct growth curves for repeated measures of length-for-age Z scores. Models that included treatment group, time since randomization, a random-effect factor for children and an interaction term for treatment by time were fitted using maximum likelihood estimation. A spatial covariance structure was used to model correlations within children. These models had a better fit (as measured by Akaike's Information Criterion) than those with compound symmetrical or unstructured correlations. A significant overall *F*-test for the treatment by time interaction at a type I error level of 0.05 was used to decide whether there was an effect of treatment on longitudinal growth. Stunting was defined as a length-for-age Z-score below -2 [24]. Because previous meta-analyses have suggested a greater response to micronutrient supplementation in stunted children, we also examined effects of supplementation in stunted children. For the main analysis second-order statistical interactions were tested between treatment group and HIV cohort over time; these were considered significant at p-value < 0.05. The growth curves modeled above were then presented graphically by fitting smoothed cubic splines to show the longitudinal trends of the supplemented arms.

### Analysis of effects on anemia

Anemia was defined as hemoglobin below 11 g/dL[25]. For this exploratory analysis, hemoglobin measurements from two time-points - the start of intervention and 12 months later - were used to determine the effects of the micronutrient supplementation. We used McNemar's test to assess within group differences in the proportion of study subjects with anemia at these two time points, and one-way ANOVA was used to assess changes in mean hemoglobin concentrations in the treatment arms. These analyses were done separately for all subjects and for those children who were anemic at baseline. Analyses of treatment effects on anemia and hemoglobin effects were confined to those subjects who had hemoglobin levels available at baseline and at 12 months.

### Statistical considerations

We estimated post-hoc power for our growth analyses bearing in mind that the numbers of children in the HIV-infected cohort were below the target determined by a-priori sample size calculations. In the cohorts of HIV-uninfected children we had adequate power using repeated measures on subjects to detect an effect size of 0.3 Z-scores, which is comparable to what had been observed in previous studies in the nutrition literature[26]. In the HIV-infected cohort, only an effect size greater than 0.5 Z-scores could be detected. For the secondary analysis on hemoglobin and anemia we assumed that there would be no meaningful difference between the cohorts of HIV-uninfected children born to HIV-infected mothers and uninfected children born to uninfected mothers so we combined these two cohorts of HIV-uninfected children in the analysis. With 65 to 70 subjects per treatment arm and conducting one-way ANOVA we had 90% power to detect differences in hemoglobin change as small as 0.3 g/dL at a 0.05 significance level. The small sample size of fourteen HIV-infected children would not allow any meaningful analyses on anemia to be conducted. The present trial was designed to detect different effects among HIV cohorts therefore analyses were stratified by HIV group. *A posteriori *exploratory analyses were also conducted to explore the differential effect of interventions on two other relevant sets of subgroups defined by stunting and diarrhea incidence, based on prior literature describing subgroup differences and biologic plausibility. The presence of these subgroup effects was evaluated by testing for statistical interaction. For these exploratory analyses second-order statistical interactions between treatment group and subgroup over time were considered significant at p-value < 0.1.

### Ethical review

The Ethics Review Committee of the University of KwaZulu-Natal and the Institutional Review Board of Tufts-New England Medical Center both approved the study. A Data Monitoring and Safety Board, established by the National Institutes of Health, monitored conduct of the trial. A parent or guardian for all children participating in the study provided written informed consent.

## Results

**Figure 1:**
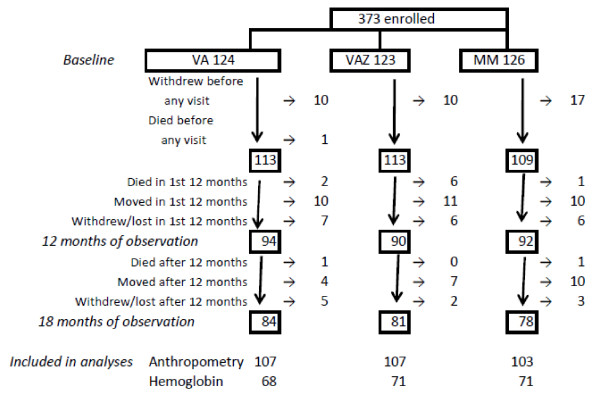
**Participant flowchart**.

Information on length Z-scores at two or more occasions was available for 317 (85%) of 373 children enrolled in the study (Figure 1): 25 HIV-infected children; 135 uninfected children born to HIV-infected mothers and 157 uninfected children born to uninfected mothers. Baseline hemoglobin measurements were available for 254 (68%) of the 373 children, baseline and 12 month hemoglobin measurements were available for 210 children (Figure 1). The treatment arms were similar in baseline characteristics (Table 1). There was no significant difference between treatment arms in the proportion of children who were lost to follow-up (including withdrawal or death). Participants were compliant with ingestion of the supplements 77% of the days they were enrolled in the study with no difference in compliance detected between treatment groups. Use of any other micronutrient supplements during the study was rare. Ten children (five in the VA arm, three in the VAZ arm and two in the MM arm) received a dose of vitamin A other than the study intervention. Fourteen children (eight in the VA arm, and three each in the other two arms) received another multivitamin during the study for an average duration of one week.

**Table 1:** Characteristics of 373 children by treatment arm

	Vitamin A (n = 124)	Vitamin A + zinc (n = 123)	Multiple micronutrients (n = 126)
HIV-infected child^δ^	9 (7.3)	11 (8.9)	12 (9.5)
HIV-uninfected child, HIV-infected mother^δ^	52 (41.9)	52 (42.3)	50 (39.7)
HIV-uninfected child, HIV-uninfected mother^δ^	63 (50.8)	60 (48.8)	64 (50.8)
Male^δ^	72 (58.1)	61 (49.6)	60 (47.6)
Length-for-age Z score*^†^	-0.65 (1.11)	-0.64 (1.27)	-0.71 (1.19)
Weight-for-age Z score*^†^	-0.06 (1.37)	0.06 (1.32)	0.05 (1.34)
Weight-for-length Z score*^†^	0.51 (1.38)	0.58 (1.30)	0.63 (1.38)
Children with stunting at baseline^δ^	26 (21.0)	20 (16.3)	23 (18.3)
Hemoglobin g/dL*	10.0 (1.29)	10.12 (1.24)	9.99 (1.19)
Duration of observation in months, median (25^th ^- 75^th ^centile)	16.6 (9.3-17.9)	16.5 (8.2-17.7)	15.7 (5.8-17.5)
Children receiving iron therapy during study period^δ^	28 (22.8)	26 (21.1)	29 (23.2)

*Values are mean (standard deviation)

δ Values are n (%)

^† ^As a Z-score of WHO International Growth References [24]

**Figure 2:**
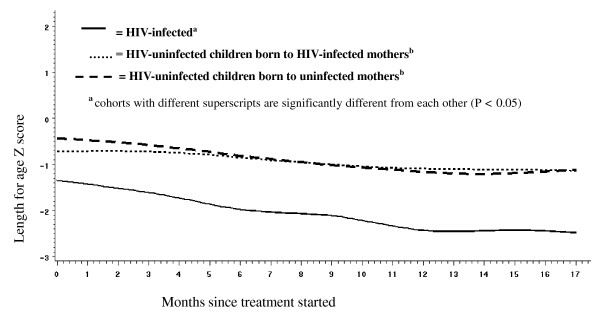
**Longitudinal changes in length-for-age Z score by HIV cohort (n = 317)**. (P = 0.001 for cohort by time interaction).

At study entry, differences in length already existed between HIV-infected and HIV-uninfected infants (Figure 2). Longitudinal growth trajectories also differed between HIV-infected compared to HIV-uninfected children, with the former showing severe declines over time (P = 0.001 for cohort by time interaction). There was also a significant statistical interaction of time, cohort and treatment (P = 0.002). HIV-uninfected children born to infected mothers and those born to uninfected mothers had similar growth trajectories during the trial. We therefore collapsed the HIV-uninfected cohorts into a single group and stratified further analyses by HIV status, namely HIV-infected versus HIV-uninfected. Among HIV-uninfected children there was no observable differential effect of treatment on length-for-age Z score trends (P = 0.37 for treatment by time interaction).

**Figure 3:**
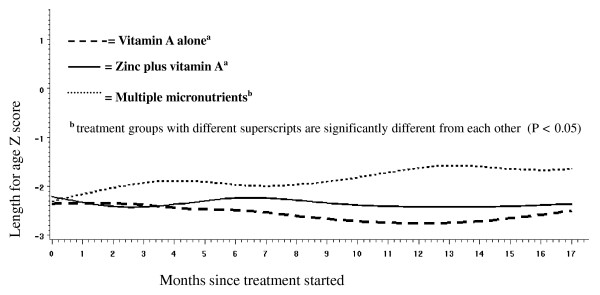
**Longitudinal changes in length-for-age Z score in children who were stunted at baseline, all cohorts combined (n = 62)**. (P = 0.029 for treatment by time interaction).

We detected an interaction between time, treatment and stunting (P = 0.08). In the 62 children in all three cohorts who were stunted at baseline the multiple micronutrient arm showed, on average, a 0.7 Z-score improvement in length-for-age Z scores over 18 months versus declines of 0.3 in vitamin A plus zinc and 0.2 in vitamin A arms. There was a significant interaction (P = 0.029) between treatment and time in stunted children (Figure 3). The pattern of improved longitudinal growth was similar when restricted to growth of 52 HIV-uninfected children with stunting at baseline, and there was also significant interaction (P = 0.027) between treatment and time. There was no detectable effect of treatment on longitudinal growth in children who were not stunted at baseline, including the cohort of 240 children who were HIV-uninfected, even after adjusting for baseline length Z-score.

**Figure 4:**
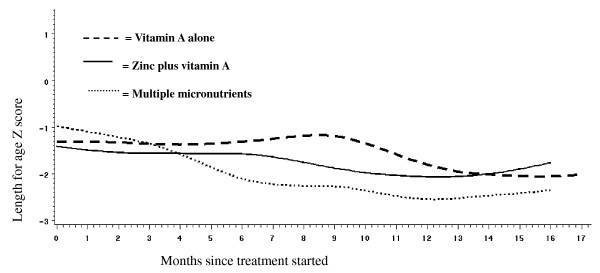
**Longitudinal changes in length-for-age Z score in HIV-infected children (n = 25)**. (P = 0.042 for treatment by time interaction).

A separate analysis of HIV-infected children found those receiving multiple micronutrient had a worse trajectory than children in the other two intervention arms (P = 0.042 for treatment by time interaction - Figure 4). Because of the small number of HIV-infected children we performed individual subject plots to detect influential subjects who might have contributed to the unexpected growth outcomes. These revealed that censorship, either due to death or loss to follow-up, was associated with preceding poor growth. Furthermore, this effect differed between treatment arms. The multiple micronutrient arm had longer follow-up per subject irrespective of the preceding growth pattern. In the other two treatment arms censorship was more likely to occur early for those subjects that had declining Z scores. In HIV-infected children mean baseline length Z-score was lower in the group of censored children (-1.84, 95% CI -2.75, -0.92) than in those who completed the study (-0.74, 95% CI -1.60, 0.12). The missing data points for HIV-infected children after loss to follow-up were therefore non-ignorable. Furthermore, HIV-infected subjects who left the study had a lower mean CD4 percentage at enrolment (15.6%, 95% CI 10.1, 21.1) than those who continued follow-up (25.6%, 95% CI 15.8 35.5). The pattern of missing data and participant attrition was not associated with preceding growth or treatment arm in HIV-uninfected children.

### Exploratory analyses of the effect of supplementation on growth among children with repeated diarrhea

**Figure 5:**
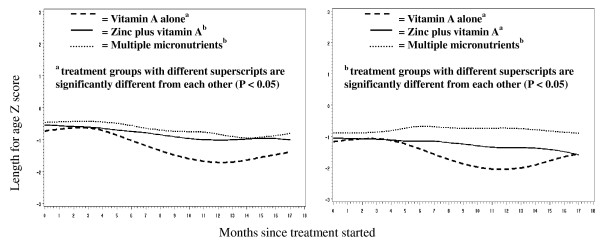
**Longitudinal changes in length-for-age Z score among HIV-uninfected children experiencing**. a) > 4 episodes of diarrhea peryear (n = 74). (P = 0.007 for treatment by time interaction). b) > 6 episodes of diarrhea per year (n = 34). (P = 0.067 for treatment by time interaction).

We examined the effects of treatment in subgroups experiencing differing diarrhea incidence. We created categories based on diarrhea rates with cut-offs being four or six episodes respectively for the high diarrhea rate group. An interaction was detected between time, treatment and diarrhea rate (P = 0.09). Multiple micronutrients supplementation and addition of zinc to vitamin A ameliorated the effects of repeated diarrhea episodes on growth in the cohorts of HIV-uninfected children (Figure 5). The protective effect of multiple micronutrients was observed in those with diarrhea incidence above four episodes per year and especially so in those experiencing more than six episodes of diarrhea per year (Figure 5). In children experiencing more than six episodes per year the multiple micronutrient arm experienced a zero Z-score change in length over time while the vitamin A and vitamin A plus zinc arms experienced 0.6 and 0.5 Z-score declines respectively (P = 0.006 for treatment by time interaction). We were unable to conduct similar analyses for HIV-infected children because of the limited number of children in this group.

### Exploratory analysis of the effect of supplementation on anemia and hemoglobin

The prevalence of anemia was 78.4% at baseline and 61.6% after 12 months respectively. In HIV-uninfected children, and in all cohorts combined, paired comparison at the two time points showed that the vitamin A and multiple micronutrient arms had significantly fewer subjects with anemia at 12 months versus baseline (Table 2). We were unable to explore changes among HIV-infected children due to limited sample size. The proportion of subjects receiving off-protocol therapeutic iron for anemia during the trial was similar across treatment arms (Table 1). Fewer children continued to be anemic at 12 months in the multiple micronutrient arm than in the other arms, but the difference between arms was not statistically significant (Table 2). For all children, combining those who were anemic and non-anemic at baseline, the vitamin A plus zinc arm tended to have a lower mean increase in hemoglobin compared to the other two arms after adjusting for cohort and baseline hemoglobin (Table 3).

**Table 2:** Proportions of study subjects with anemia^1 ^at baseline and 12 months by HIV status and treatment arm

Cohort	Treatment arms	n	Subjects with anemia n (%)^2^		
			**Baseline**	**12 months**	**P value†**
HIV-uninfected (n = 196)	Vitamin A only	64	49 (76.6)	38 (59.4)	0.0278
	Vitamin A + zinc	67	51 (76.1)	42 (62.7)	0.0833
	Multiple micronutrients	65	54 (83.1)	38 (58.5)	0.0017
All cohorts (n = 210)	Vitamin A only	68	52(76.5)	40 (58.8)	0.0186
	Vitamin A + zinc	71	54 (76.1)	46 (64.8)	0.1306
	Multiple micronutrients	71	60 (84.5)	43 (60.6)	0.0011

^1 ^Anemia is defined as hemoglobin level below 11 g/dL [25]

^2 ^Analysis includes subjects with hemoglobin available at baseline and twelve months

†Within group changes from baseline (McNemar Test)

**Table 3:** Change in hemoglobin in all subjects stratified by presence of anemia at baseline

	n	Mean change in hemoglobin g/dL, mean (95% CI)^1 ^[n per treatment arm]			
		**Vitamin A only**	**Vitamin A + zinc**	**Multivitamins**	**Overall P^2^**
**All subjects**	210	0.59 (0.22, 0.97)^b ^[68]	0.17 (-0.20, 0.54)^a ^[71]	0.62 (0.26, 0.98)^b ^[71]	0.06
**Anemic** **at baseline**	166	0.99 (0.56, 1.41) [52]	0.49 (0.07, 0.91) [54]	0.93 (0.54, 1.31) [60]	0.10
**Not anemic**	44	-0.92 (-1.81, -0.02) [16]	-1.12 (-2.00, -0.25) [17]	-0.48 (-1.56, 0.59) [11]	0.49

^1 ^Adjusted for baseline hemoglobin and cohort

^2 ^ANOVA F-test

Values in the same row with different superscripts are significantly different from each other (*P < 0.05*)

## Discussion

Daily multiple micronutrient supplementation that included vitamin A and zinc, when compared to vitamin A alone or to vitamin A plus zinc, improved longitudinal growth over 18 months in children who were stunted at baseline. This is in keeping with the findings of a meta-analysis that found that multiple micronutrients, but not vitamin A or iron alone, improved longitudinal growth in children[26]. Previous findings [7] in conjunction with the present study suggest that the failure of zinc alone, or zinc and vitamin A, to improve growth may be related to the coexistence of other nutrient deficiencies, such as iron, thus limiting the response to zinc supplementation when used alone. This is further supported by a trial in Chinese children that found multiple micronutrients with zinc to be better than zinc alone in improving growth velocity[27]. The differences across trials in various geographic regions may be attributable to differences in prevalence of stunting, iron and other micronutrient deficiencies (such as vitamin D), or to infectious disease burden and spectrum.

Exploratory analyses found that multiple micronutrients, when compared to vitamin A alone or with zinc, ameliorated the effect of recurrent diarrhea on stunting. Zinc has been shown to be effective in the case management of diarrhea - reducing the duration of the diarrhea episode and improving growth thereafter[28]. To our knowledge the protective effect of zinc, or multiple micronutrients, against the growth retarding effects of repeated episodes of diarrhea has not been demonstrated in previous trials. The finding from this study that multiple micronutrient supplementation ameliorates the effect of diarrhea on growth suggests that progressive stunting in children with repeated diarrhea may be related to deficiencies of multiple nutrients. This finding, arising from exploratory analyses, needs further investigation in larger studies.

The relatively small number of HIV-infected children in the study, and the non-ignorable pattern of missing data in this group, limits our ability to detect effects and make inferences for the cohort of HIV-infected children. All HIV-infected children with available CD4 counts who dropped out of the study either due to death or loss to follow-up had severe HIV disease based on immunologic criteria. The worse growth pattern seen in the multiple micronutrient arm is thus most likely related to the longer follow-up of stunted children in this arm rather than any impact of a particular micronutrient formulation. One may anticipate better cohort retention and very different outcomes in the presence of antiretroviral therapy. Our findings emphasize the need for evaluating how patterns of missing data and severity stratification may influence outcomes in trials involving HIV-infected children.

While all three treatment arms had reductions in the proportion of children with anemia over 12 months, supplementation with multiple micronutrients tended to achieve the greatest reduction in anemia. This is supported by findings from a large multi-centre trial of multiple micronutrients that included South African[5]; Vietnamese [11]; Indonesian [29]; and Peruvian infants[9]. In the Indonesian trial daily supplementation with multiple micronutrients was better than supplementation with iron alone in improving the hemoglobin of anemic infants. This occurred despite a lack of effect of multiple micronutrients in reducing morbidity or improving growth.

In this trial, anemia and hemoglobin change were not included as *a priori *hypotheses. Children with a hemoglobin level below 10 g/dL received iron treatment while continuing the study intervention. We were unable to examine whether the micronutrients prevented anemia because there was already a high prevalence of anemia at baseline. While similar numbers of children in each arm received iron treatment, fewer children in the multiple micronutrient arm remained anemic at the end of 12 months compared to the other arms. This may be due to the presence of iron in the multi-micronutrient formulation, resulting in an effectively higher total daily dose of iron than what would have been ingested through the therapeutic iron alone. The relatively smaller increment in hemoglobin in the vitamin A plus zinc arm compared to vitamin A alone suggests a possible inhibitory effect of zinc on iron absorption. The multiple micronutrient arm showed similar improvements in mean hemoglobin to the vitamin A arm despite containing zinc. It is possible that the additional micronutrients alleviated the negative effect of co-administration of iron and zinc. This is an important observation, albeit arising from exploratory analyses, that a combination of micronutrients may improve childhood morbidity while minimizing the potential negative biologic interactions between these nutrients.

The effect of the micronutrient supplements on anemia and hemoglobin were not among the primary aims of the study, and thus the results of this analysis, as with any post-hoc analysis, have to be interpreted with caution. While similar numbers of children in each treatment arm received therapeutic iron the possibility of unmeasured confounding still remains. However, we think that this may improve generalizability to programmatic conditions where anemic children would receive iron therapy as standard of care. This is especially relevant to our trial, where children were enrolled for 18 months and exposure to other interventions - such as supplemental iron - is likely under non-trial conditions. The trial of zinc and multiple micronutrients in Peru by Penny et al. [30] also reported out-of-protocol administration of therapeutic iron to large numbers of subjects in whom anemia was detected. Interestingly, that study also found a pattern of lower hemoglobin responses in the group that received zinc only as opposed to group who received zinc plus multiple micronutrients.

## Conclusions

In summary, these findings suggest that daily multiple micronutrients added to vitamin A supplementation in infants and children may be useful in improving growth in children who are stunted or who have repeated diarrhea, and thus as a public health intervention in communities with a high prevalence of either stunting and recurrent diarrhea. The significant effect of multiple micronutrients compared to vitamin A alone that was the control group, or to vitamin A plus zinc, on longitudinal growth in this study is important for other African countries where zinc supplementation alone was not found to reverse stunting. The question of individual targeting for stunted children versus universal administration of multiple micronutrients to children in high-risk environments still needs to be resolved. Further evidence is needed to confirm the beneficial effects of micronutrients on anemia, and to determine whether similar benefits apply to HIV-infected children - especially those receiving antiretroviral therapy, which is now the standard of care. While exploratory analyses suggest an inhibitory effect of zinc on iron absorption, the co-administration of other micronutrients may negate this inhibitory effect.

## Competing interests

The authors declare that they have no competing interests.

## Authors' contributions

MKC designed and conducted the analyses and wrote the initial draft of the manuscript. JVdB and KAL served as project directors. MLB and AT were responsible for study design. NM supervised fieldwork. The manuscript was critically reviewed and approved by all of the authors.

## Pre-publication history

The pre-publication history for this paper can be accessed here:

http://www.biomedcentral.com/1471-2458/10/145/prepub

## References

[B1] World Health Organization global database on child growth and nutrition http://www.who.int/nutgrowthdb/database/en/

[B2] SAVACG The South African Vitamin A Consulting Group Survey: Children aged 6-71 months in South Africa 1994: their anthropometric, vitamin A, iron and immunisation coverage and status 1995 8693371

[B3] Labadarios D Steyn NP Maunder E MacIntryre U Gericke G Swart R Huskisson J Dannhauser A Vorster HH Nesmvuni AE The National Food Consumption Survey (NFCS): South Africa, 1999 Public Health Nutr 2005 10 5 533 543 10.1079/PHN2005816 16153334

[B4] Smuts CM Lombard CJ Benade AJ Dhansay MA Berger J Hop le T Lopez de Romana G Untoro J Karyadi E Erhardt J Efficacy of a foodlet-based multiple micronutrient supplement for preventing growth faltering, anemia, and micronutrient deficiency of infants: the four country IRIS trial pooled data analysis J Nutr 2005 10 3 631S 638S 15735107 10.1093/jn/135.3.631S

[B5] Smuts CM Dhansay MA Faber M van Stuijvenberg ME Swanevelder S Gross R Benade AJ Efficacy of multiple micronutrient supplementation for improving anemia, micronutrient status, and growth in South African infants J Nutr 2005 10 3 653S 659S 15735110 10.1093/jn/135.3.653S

[B6] Brown KH Peerson JM Rivera J Allen LH Effect of supplemental zinc on the growth and serum zinc concentrations of prepubertal children: a meta-analysis of randomized controlled trials Am J Clin Nutr 2002 10 6 1062 1071 12036814 10.1093/ajcn/75.6.1062

[B7] Muller O Garenne M Reitmaier P Van Zweeden AB Kouyate B Becher H Effect of zinc supplementation on growth in West African children: a randomized double-blind placebo-controlled trial in rural Burkina Faso International journal of epidemiology 2003 10 6 1098 1102 10.1093/ije/dyg190 14681282

[B8] Allen LH Iron supplements: scientific issues concerning efficacy and implications for research and programs J Nutr 2002 10 4 Suppl 813S 819S 11925487 10.1093/jn/132.4.813S

[B9] Lopez de Romana G Cusirramos S Lopez de Romana D Gross R Efficacy of multiple micronutrient supplementation for improving anemia, micronutrient status, growth, and morbidity of Peruvian infants J Nutr 2005 10 3 646S 652S 15735109 10.1093/jn/135.3.646S

[B10] Allen LH Rosado JL Casterline JE Lopez P Munoz E Garcia OP Martinez H Lack of hemoglobin response to iron supplementation in anemic mexican preschoolers with multiple micronutrient deficiencies Am J Clin Nutr 2000 10 6 1485 1494 10837289 10.1093/ajcn/71.6.1485

[B11] Hop le T Berger J Multiple micronutrient supplementation improves anemia, micronutrient nutrient status, and growth of Vietnamese infants: double-blind, randomized, placebo-controlled trial J Nutr 2005 10 3 660S 665S 15735111 10.1093/jn/135.3.660S

[B12] Thu BD Schultink W Dillon D Gross R Leswara ND Khoi HH Effect of daily and weekly micronutrient supplementation on micronutrient deficiencies and growth in young Vietnamese children Am J Clin Nutr 1999 10 1 80 86 9925127 10.1093/ajcn/69.1.80

[B13] Geltman PL Meyers AF Mehta SD Brugnara C Villon I Wu YA Bauchner H Daily multivitamins with iron to prevent anemia in high-risk infants: a randomized clinical trial Pediatrics 2004 10 1 86 93 10.1542/peds.114.1.86 15231912

[B14] Henderson RA Saavedra JM Perman JA Hutton N Livingston RA Yolken RH Effect of enteral tube feeding on growth of children with symptomatic human immunodeficiency virus infection J Pediatr Gastroenterol Nutr 1994 10 4 429 434 8071777 10.1097/00005176-199405000-00004

[B15] Kreuzer KA Rockstroh JK Jelkmann W Theisen A Spengler U Sauerbruch T Inadequate erythropoietin response to anaemia in HIV patients: relationship to serum levels of tumour necrosis factor-alpha, interleukin-6 and their soluble receptors Br J Haematol 1997 10 2 235 239 10.1046/j.1365-2141.1997.d01-2031.x 9029005

[B16] Arpadi SM Growth failure in children with HIV infection Journal of Acquired Immune Deficiency Syndromes 2000 10 Suppl 1 S37 42 10.1097/00042560-200010001-00006 11126424

[B17] Luabeya KK Mpontshane N Mackay M Ward H Elson I Chhagan M Tomkins A Broeck J Van den Bennish ML Zinc or multiple micronutrient supplementation to reduce diarrhea and respiratory disease in South african children: a randomized controlled trial PLoS ONE 2007 10 6 e541 10.1371/journal.pone.0000541 17593956 PMC1891438

[B18] Chhagan MK Broeck J Van den Luabeya KK Mpontshane N Tucker KL Bennish ML Effect of micronutrient supplementation on diarrhoeal disease among stunted children in rural South Africa EJCN 2009 1 8 19174830 10.1038/ejcn.2008.78 PMC2705811

[B19] Bland RM Little KE Coovadia HM Coutsoudis A Rollins NC Newell ML Intervention to promote exclusive breast-feeding for the first 6 months of life in a high HIV prevalence area AIDS 2008 10 7 883 891 10.1097/QAD.0b013e3282f768de 18427207

[B20] Mpontshane N Broeck J Van den Chhagan M Luabeya KK Johnson A Bennish ML HIV infection is associated with decreased dietary diversity in South African children J Nutr 2008 10 9 1705 1711 18716173 10.1093/jn/138.9.1705 PMC2587082

[B21] Broeck J Van den Mackay M Mpontshane N Kany Kany Luabeya A Chhagan M Bennish ML Maintaining data integrity in a rural clinical trial Clinical trials (London, England) 2007 10 5 572 582 17942472 10.1177/1740774507084106

[B22] Hamill PV Drizd TA Johnson CL Reed RB Roche AF Moore WM Physical growth: National Center for Health Statistics percentiles Am J Clin Nutr 1979 10 3 607 629 420153 10.1093/ajcn/32.3.607

[B23] Anthropometric standardization Growth Analyser, version 3.0 Dutch Growth Foundation, Rotterdam http://www.growthanalyser.org

[B24] The World Health Organization child growth standards http://www.who.int/childgrowth/en/

[B25] WHO Indicators and strategies for iron deficiency and anaemia programmes 1994 Geneva: World Health Organization

[B26] Ramakrishnan U Aburto N McCabe G Martorell R Multimicronutrient interventions but not vitamin a or iron interventions alone improve child growth: results of 3 meta-analyses J Nutr 2004 10 10 2592 2602 15465753 10.1093/jn/134.10.2592

[B27] Sandstead HH Penland JG Alcock NW Dayal HH Chen XC Li JS Zhao F Yang JJ Effects of repletion with zinc and other micronutrients on neuropsychologic performance and growth of Chinese children Am J Clin Nutr 1998 10 2 Suppl 470S 475S 9701162 10.1093/ajcn/68.2.470S

[B28] Roy SK Tomkins AM Akramuzzaman SM Chakraborty B Ara G Biswas R Islam KE Khatun W Jolly SP Impact of zinc supplementation on subsequent morbidity and growth in Bangladeshi children with persistent diarrhoea Journal of health, population, and nutrition 2007 10 1 67 74 17615905 PMC3013265

[B29] Untoro J Karyadi E Wibowo L Erhardt MW Gross R Multiple micronutrient supplements improve micronutrient status and anemia but not growth and morbidity of Indonesian infants: a randomized, double-blind, placebo-controlled trial J Nutr 2005 10 3 639S 645S 15735108 10.1093/jn/135.3.639S

[B30] Penny ME Marin RM Duran A Peerson JM Lanata CF Lonnerdal B Black RE Brown KH Randomized controlled trial of the effect of daily supplementation with zinc or multiple micronutrients on the morbidity, growth, and micronutrient status of young Peruvian children Am J Clin Nutr 2004 10 3 457 465 14985222 10.1093/ajcn/79.3.457

